# Modified Distribution Entropy as a Complexity Measure of Heart Rate Variability (HRV) Signal

**DOI:** 10.3390/e22101077

**Published:** 2020-09-24

**Authors:** Radhagayathri Udhayakumar, Chandan Karmakar, Peng Li, Xinpei Wang, Marimuthu Palaniswami

**Affiliations:** 1 School of Information Technology, Deakin University, 75 Pigdons Road, Waurn Ponds, Geelong, VIC 3216, Australia; radhagayathri.udhayakumar@deakin.edu.au; 2 Division of Sleep and Circadian Disorders, Brigham and Women’s Hospital, Harvard Medical School, Boston, MA 02115, USA; pli9@bwh.harvard.edu; 3 School of Control Science and Engineering, Shandong University, Jinan 250100, China; wangxinpei@sdu.edu.cn; 4 Department of Electrical & Electronic Engineering, The University of Melbourne, Melbourne, VIC 3010, Australia; palani@unimelb.edu.au

**Keywords:** distribution entropy, complexity analysis, heart rate variability, Shannon entropy

## Abstract

The complexity of a heart rate variability (HRV) signal is considered an important nonlinear feature to detect cardiac abnormalities. This work aims at explaining the physiological meaning of a recently developed complexity measurement method, namely, distribution entropy (DistEn), in the context of HRV signal analysis. We thereby propose modified distribution entropy (mDistEn) to remove the physiological discrepancy involved in the computation of DistEn. The proposed method generates a distance matrix that is devoid of over-exerted multi-lag signal changes. Restricted element selection in the distance matrix makes “mDistEn” a computationally inexpensive and physiologically more relevant complexity measure in comparison to DistEn.

## 1. Introduction

Heart rate variability (HRV) analysis is a powerful non-invasive method used to examine the functioning of the autonomic nervous system (ANS). It is useful to understand the interplay between the sympathetic and parasympathetic wings of ANS that serve to speed up and slow down the heart rate respectively [1]. HRV, a variation of the time period between consecutive heart beats (RR intervals), is thought to reflect the heart’s adaptability to changing physiological conditions. Various HRV measures are considered to be critical bio-markers for understanding and diagnosing cardiac health [2,3]. Popular non-linear entropy statistics such as ApEn and SampEn are significant bio-markers that measure the extent of irregularities contained in HRV signals [4,5,6]. Physiological signals are highly non-linear in nature, so it is important to use non-linear tools of analysis over the linear ones [7,8,9,10].

The functioning of a healthy cardiac system is associated with higher complexity than one with some sort of cardiac ailment. A high level of complexity does not necessarily indicate a high level of irregularity [11]. ApEn and SampEn, being measures of irregularity [12,13], do not always translate to the level of complexity contained in the underlying system. ApEn and SampEn assess a signal’s state of orderliness (or chaos) by surveying existential patterns interpreted from the signal. An irregular signal may not always be associated with a high level of complexity and vice versa. For example, when an original time series (say, one that represents an underlying complex system) is randomized to form its surrogate time series, ApEn or SampEn will be higher for the surrogate series than the original. However, is this increase in randomness (or entropy) also a reflection of increase in complexity of the representative system? No, because technically, randomization breaks the inherent structure of the originally complex series, leading to information loss, in other words a loss of content/complexity [14]. Many previous studies have reported higher irregularity in arrhythmic cardiac signals than their healthy counterparts [11,15]. However, an arrhythmic heart functions with a much lower level of complexity than a healthy one. In such a case, analyzing complexity apart from irregularity becomes very significant.

Distribution entropy (DistEn) is a recently introduced measure of signal “complexity”. It is calculated from the empirical probability distribution function (ePDF) of vector-to-vector distances of the signal [16]. DistEn has been used to extract complexity information (rather than irregularity) from HRV signals [16,17,18]. DistEn follows the same conceptual strategy as ApEn and SampEn. However, unlike ApEn or SampEn, DistEn (1) quantifies complexity, not irregularity and (2) is computationally superior, since it does not require use of the most critical [4,19] parameter *r* (tolerance) like ApEn or SampEn do [16].

DistEn is a function of three parameters:m data length *N*, embedding dimension *m* and number of bins *M* used in the probability distribution. In most cases, DistEn is known to be less influenced by changes in *N* ad *M* [16,20]. Additionally, DistEn performs better than other entropy measures, especially for short length signals [16]. DistEn’s efficiency as a complexity measure and bio-marker has been tested and proved good in the cases of both synthetic and physiological signals [16].

In this study, we explore the physiological relevance of DistEn in HRV analysis. We hypothesized that such an exploration could answer significant questions. For instance: (1) Is the quantified DistEn value a direct consequence of any underlying physiological mechanism? (2) In DistEn measurement, can the distance between template vectors be mapped to change in a physiological factor? Consequently, we introduce a variant of DistEn; “modified distribution entropy (mDistEn),” which is defined considering the underlying physiology of a HRV signal. Finally, the efficacy of mDistEn is compared to that of DistEn, as a bio-marker of cardiac health.

The novelty of this modified algorithm lies in the way mDistEn takes advantage of the distances between vectors within a certain time lag instead of collecting the distances across all vectors in the state space, the way original DistEn does.

## 2. Data and Methods

### 2.1. Data

1. Synthetic: Logistic time series at two different levels of irregularity were used for the study. The data were generated using the logistic map $x_{n+1}=ax_{n}\left(1-x_{n}\right)$ using MATLAB R2019b. The initial value $x_{n}$ was set as 0.5. The constant *a* represents the level of irregularity in the generated signal; a=3.5 for a “periodic” time-series and a=4 for a “chaotic” one. While generating the time-series, the function also adds a random noise to the signal as follows: $X_{logistic}=x_{n+1}+x_{noise}$, where $x_{noise}=\left[x_{random}*noiseLevel*SD\left(x_{n+1}\right)\right]$. Here $x_{random}$ is a normally distributed signal of random numbers, of the same length as $x_{n+1}$. The noiseLevel (noise standard deviation divided by the standard deviation of the noise-free time series) of the function is set at 0.1. SD represents the standard deviation. Ten different realizations (difference being created by the new random noise added each time) were synthesized at each level of irregularity, namely, “periodic” and “chaotic.” We only used logistic map to produce time-series with chaotic and periodic regimes since it has been the simplest and most widely used on synthetic data examples to demonstrate entropy level variations [5,16,21,22,23]. Data lengths of 50, 100, 200, 500 and 1000 were used for the generation.
2. Physiological: All real time RR interval data were obtained from the PhysioNet database [24]. Corrected beat annotation files were available from the database. These were further manually corrected to remove the ectopic beats. The data included: (i) Healthy: RR interval time-series of 72 normal sinus rhythm subjects were obtained from PhysioNet, which included 18 subjects from the MIT-BIH Normal Sinus Rhythm database (nsrdb) and 54 subjects from Normal Sinus Rhythm RR Interval database (nsr2db). (ii) Diseased: RR interval time-series of diseased subjects were obtained from the MIT-BIH database of PhysioNet, constituting (a) 48 arrhythmic data extracted from 47 subjects [25]. The recordings were digitized at 360 samples per second per channel with 1-bit resolution over a 10 mV range; (b) 25 atrial fibrillated data [25], each sampled at 250 samples per second with 12-bit resolution over a range of 10 millivolts. Atrial fibrillation is a specific category of arrhythmia related to paroxysmal atrial malfunctions. Atrial fibrillation is the most common form of arrhythmia and can occur as a post-surgical event, unlike many other common arrhythmias. After direct extraction of RR interval series from all data, each signal segment was selected from the beginning by varying length from 50 to 1000 (total 5 different lengths—50, 100, 200, 500 and 1000 beats).

### 2.2. Distribution Entropy

Distribution entropy (DistEn) is calculated based on the empirical probability distribution function (ePDF) of distances among vectors formed from a given time series [16]. For given time series data $\left\{x(n):1\leq n\leq N\right\}$ of length *N* and embedding dimension *m*, DistEn is calculated as follows:

1. Form (N−m) vectors of length *m* each, given by
  $$\left\{X_{i}^{m}:1\leq i\leq \left(N-m\right)\right\}$$
  where
  (1) $$X_{i}^{m}=\left\{x(i+k):0\leq k\leq m-1\right\}$$
2. Take each $X_{i}^{m}$ vector of step 1 as a template vector and find its distance from every vector $X_{j}^{m}$, where the distance is given by
  (2) $$d_{ij}^{m}=\{\max|X_{i}^{m}-X_{j}^{m}\left|:\;1\leq j\leq (N-m),\;j\neq i\right\}$$
3. This when repeated for all *i*-th template vectors where 1≤i≤(N−m), a distance matrix *D* of dimension (N−m)*(N−m−1) is formed as shown below
  (3) $$D=\left[\begin{array}{cccc} d_{12}^{m} & d_{13}^{m} & \cdots & d_{1(N-m)}^{m}\\ d_{21}^{m} & d_{23}^{m} & \cdots & d_{2(N-m)}^{m}\\ \vdots & \vdots & \cdots & \vdots \\ \vdots & \vdots & \cdots & d_{\left(N-m-1)(N-m\right)}^{m}\\ d_{(N-m)1}^{m} & d_{(N-m)2}^{m} & \cdots & d_{\left(N-m)(N-m-1\right)}^{m} \end{array}\right]$$
4. From matrix (3), it is evident that elements in *D* are being repeated twice, i.e., $d_{ij}^{m}=d_{ji}^{m}$. This is true because the distances are absolute values as can be seen from Equation (2). Thus, in formulating DistEn, it becomes sufficient to use either the upper triangle or lower triangle of *D* [16]. Here, we use the upper triangle only and denote the resulting matrix as $D^{{}^{\prime }}$, where
  (4) $$D^{{}^{\prime }}=\left[\begin{array}{ccccc} d_{12}^{m} & d_{13}^{m} & \cdots & \cdots & d_{1(N-m)}^{m}\\ \; & d_{23}^{m} & d_{24}^{m} & \cdots & d_{2(N-m)}^{m}\\ \; & \; & d_{34}^{m} & \cdots & d_{3(N-m)}^{m}\\ \; & \; & & \; & \vdots \\ \; & \; & & \; & \vdots \\ \; & \; & & \; & d_{\left(N-m-1)(N-m\right)}^{m} \end{array}\right]$$
5. The elements of distance matrix $D^{{}^{\prime }}$ are now divided equally into *M* number of bins and the corresponding histogram is obtained.
6. Now, at each bin *t* of the histogram, its probability is estimated as
  (5) $$p_{t}=\frac{\mathrm{count}\;\mathrm{in}\;\mathrm{bin}\;t}{\mathrm{total}\;\mathrm{number}\;\mathrm{of}\;\mathrm{elements}\;\mathrm{in}\;\mathrm{matrix}\;D}$$
  for 1≤t≤M. $p_{t}$ is the probability of the *i*-th bin in the histogram.
7. By the definition of Shannon entropy, the normalized DistEn of a given time series x(n) is defined by the expression
  (6) $$DistEn\left(m,M\right)=\begin{array}{ccc} \; & M\\ \frac{-1}{\log_{2}\left(M\right)} & \sum & p_{t}\log_{2}\left(p_{t}\right)\\ \; & t=1 \end{array}$$

### 2.3. Modified Distribution Entropy

#### 2.3.1. Physiological Explanation of Distance$d_{ij}^{m}$ in DistEn Measurement for HRV Signal

Let an inter-heartbeat RR interval time series of length *N* be defined as

(7) $$\begin{array}{cccccc} RR=\{ & RR_{1} & RR_{2} & RR_{3} & \ldots & RR_{N} \end{array}\}$$

For an embedding dimension *m*, (N−m) template vectors can be defined using Equation (1) and for m=1 the template vectors of RR will be:

(8) $$\begin{array}{c} X_{1}^{1}=RR_{1},X_{2}^{1}=RR_{2},X_{3}^{1}=RR_{3},\\ \ldots \ldots X_{\left(N-1\right)}^{1}=RR_{\left(N-1\right)} \end{array}$$

**Figure 1:**
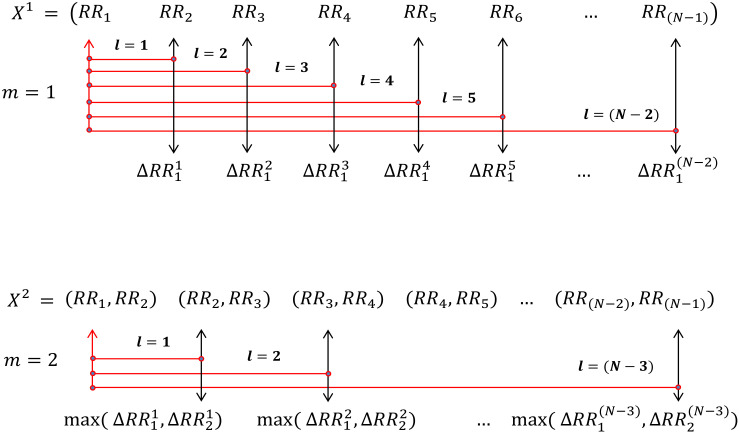
Changes of individual RR intervals from their *l* lagged RR interval for embedding dimension m=1,2.

Now, the distance of vectors $\left\{X_{j}^{1}|2\leq j\leq N-1\right\}$ from template vector $X_{1}^{1}$ can be computed using Equation (2) as follows:

(9) $$\begin{array}{c} d_{12}^{1}=|X_{1}^{1}-X_{2}^{1}|=max(|RR_{1}-RR_{2}\left|\right)\\ =|RR_{1}-RR_{2}|=\Delta RR_{1}^{1}\\ d_{13}^{1}=|X_{1}^{1}-X_{3}^{1}|=max(|RR_{1}-RR_{3}\left|\right)\\ =|RR_{1}-RR_{3}|=\Delta RR_{1}^{2}\\ \cdots \\ d_{1(N-1)}^{1}=|X_{1}^{1}-X_{\left(N-1\right)}^{1}|=max(|RR_{1}-RR_{N-1}\left|\right)\\ =|RR_{1}-RR_{N-1}|=\Delta RR_{1}^{N-2} \end{array}$$

where $\Delta RR_{i}^{l}=\left|RR_{i}-RR_{i+l}\right|$ and *i* denotes the *i*-th RR interval and *l* is the lag or delay used to calculate the change between RR intervals (shown in Figure 1). Similarly, for embedding dimension m=2, the template vectors can be defined as:

(10) $$\begin{array}{c} X_{1}^{2}=\left(RR_{1},RR_{2}\right),X_{2}^{2}=\left(RR_{2},RR_{3}\right),\\ X_{3}^{2}=\left(RR_{3},RR_{4}\right),\\ \ldots X_{\left(N-2\right)}^{2}=\left(RR_{\left(N-2\right)},RR_{\left(N-1\right)}\right) \end{array}$$

Now, the distance of vectors $\left\{X_{j}^{2}|2\leq j\leq N-2\right\}$ from template vector $X_{1}^{2}$ can be computed using Equation (2) as follows:

(11) $$\begin{array}{c} d_{12}^{2}=\\ |X_{1}^{2}-X_{2}^{2}|=max(|RR_{1}-RR_{2}|,|RR_{2}-RR_{3}\left|\right)\\ =max(\Delta RR_{1}^{1},\Delta RR_{2}^{1})\\ d_{13}^{2}=\\ |X_{1}^{2}-X_{3}^{2}|=max(|RR_{1}-RR_{3}|,|RR_{2}-RR_{4}\left|\right)\\ =max(\Delta RR_{1}^{2},\Delta RR_{2}^{2})\\ \cdots \\ d_{1(N-2)}^{2}=\\ |X_{1}^{2}-X_{\left(N-2\right)}^{2}|=max(|RR_{1}-RR_{N-2}|,|RR_{2}-RR_{N-1}\left|\right)\\ =max(\Delta RR_{1}^{N-3},\Delta RR_{2}^{N-3}) \end{array}$$

This signifies that $d_{ij}^{2}$ quantifies the maximum of changes of individual RR interval from its *l*(1≤l≤N−m−1) lagged or delayed RR interval for embedding dimension m=2 (shown in Figure 1). Therefore, the generalized distance Equation (2) can be rewritten with respect to RR interval signal as:

(12) $$\begin{array}{cc} d_{ij}^{m} & =\left\{max\left(\Delta RR_{i}^{l},\Delta RR_{i+1}^{l},\ldots ,\Delta RR_{i+m-1}^{l}\right\}\right)\\ \; & :\;1\leq i,j\leq (N-m),\;j\neq i,l=|i-j|\} \end{array}$$

Therefore, DistEn is a measure of the Shannon entropy of change of an RR interval calculated for lags ranging from 1:(N−m−1). The embedding dimension *m* controls the calculation of change by defining the number of candidates for maximum change calculation.

#### 2.3.2. Elimination of lags>10

From the analytical explanation of DistEn, it is obvious that it measures the entropy of the change or the derivative of the HRV signal at all lags 1:(N−m−1). Therefore, the maximum lag at which the change is measured depends on the data length *N* and embedding dimension *m*. Since N≫m, we can say that the maximum lag predominantly depends on the length of the signal. The physiological discrepancy in defining DistEn lies behind this dependency of lag on data length. If we consider the physiological mechanism of heart rate variability, the effect of the present heart beat on future heart beats is defined by the properties of cardiovascular mechanisms rather than recording length or number of heart beats. Therefore, the use of lags based on data length (for calculating change in HRV) may mostly assess random phenomena rather than physiological information. In previous studies, it has been reported that a heartbeat’s influence is felt on an average of only 6–10 beats following it [26,27]. Thus it becomes physiologically irrelevant to find the change between a given beat and all other beats following it, as is done in the case of DistEn. Thus, from $D^{{}^{\prime }}$, it is physiologically justified to remove all changes corresponding to lags >10. This modification to $D^{{}^{\prime }}$ results in $D^{{}^{\prime \prime }}.$

(13) $$\begin{array}{cc} \; & \begin{array}{c} D^{{}^{\prime \prime }}= \end{array}\\ \; & \left[\begin{array}{cccccc} d_{12} & d_{13} & \cdots & d_{1(11)}\\ \; & d_{23} & d_{24} & \cdots & d_{2(12)}\\ \; & \; & d_{34} & \cdots & \cdots \\ \; & \; & & d_{46} & \cdots \\ \; & \; & & \; & & d_{\left(N-m-10)(N-m\right)}\\ \; & \; & & \; & & \vdots \\ \; & \; & & \; & & d_{\left(N-m-1)(N-m\right)} \end{array}\right] \end{array}$$

This modified distance matrix $D^{{}^{\prime \prime }}$ (13) is now subjected to Shannon entropy calculation using steps 5 to 7 of Section 2.2 for evaluation of modified distribution entropy (mDistEn) of the signal.

### 2.4. Statistical Analysis

In order to test the efficiency of regularity measures as classification features, we need to find their strength in separating data belonging to different classes. In our study, we have used the statistical test parameters *p* and AUC for the purpose. The *p*-value obtained using Mann–Whitney U test represents the probability of X and Y belonging to continuous distributions of the same median, where X and Y are samples taken from two independent populations. *p* can take values from 0 to 1 and in this study we have considered p< 0.05 as statistical significance. AUC, the area under the ROC (receiver operating characteristic) curve is the probability that a classifier ranks a randomly chosen instance X higher than a randomly chosen instance Y—X and Y being samples taken from two independent populations. An AUC value of 0.5 indicates that the distributions of the features are similar in the two groups with no discriminatory power. Conversely, an ROC area value of 1.0 would mean that the distributions of the features of the two groups do not overlap at all. The statistics toolbox of MATLAB R2019b was used to perform all statistical tests.

## 3. Results

### 3.1. Effect of Eliminating lags>10 from $D^{{}^{\prime }}$

**Figure 2:**
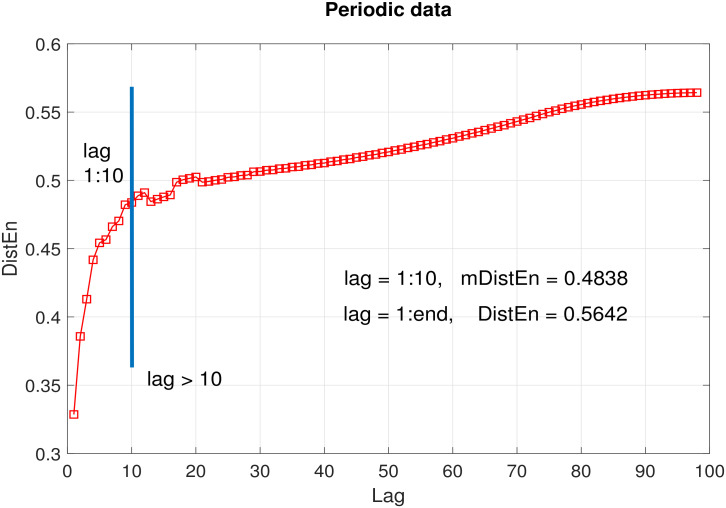
Average DistEn of periodic data (10 realizations) as a function of lag. Blue line indicates the end of first 10 lags. mDistEn calculated using the first 10 lags was 0.4838, while DistEn calculated using all lags was 0.5642.

**Figure 3:**
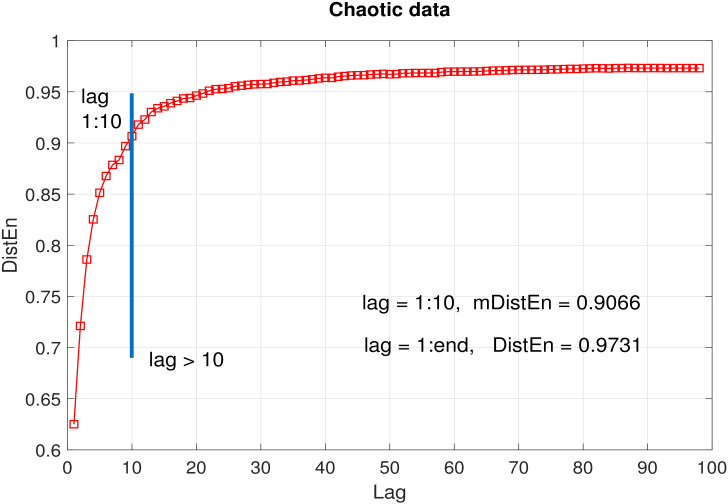
Average DistEn of chaotic data (10 realizations) as a function of lag. Blue line indicates the end of first 10 lags. mDistEn calculated using the first 10 lags was 0.9066, while DistEn calculated using all lags was 0.9731.

**Figure 4:**
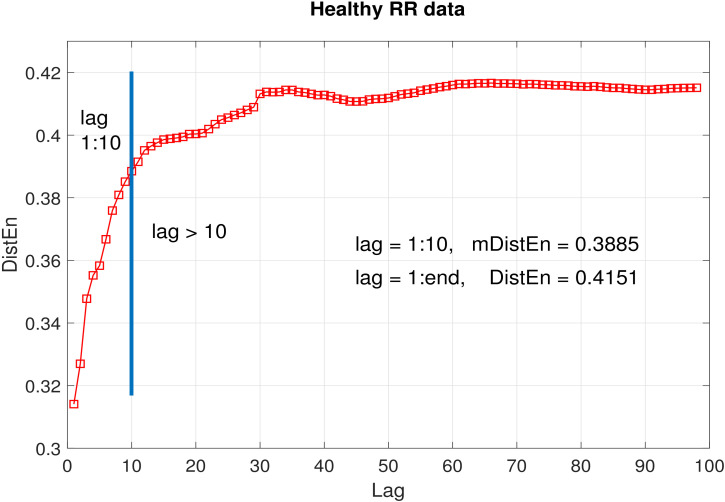
Average DistEn of healthy RR interval data (72 RR interval time-series) as a function of lag. Blue line indicates the end of first 10 lags. mDistEn calculated using the first 10 lags was 0.0.3885, while DistEn calculated using all lags was 0.4151.

For a data of length N=100, the average DistEn was calculated for each lag *l* ranging from 1 to 99; the histogram consisted of elements of *D* corresponding to lags 1:*l*. The embedding dimension value was 2 and the value of parameter *M* wass kept fixed at 500. As can be seen from Figure 2, Figure 3 and Figure 4, the entropy values obtained using lags from 1 to 10 (i.e., mDistEn) were 0.4838, 0.9066 and 0.3885 (marked by a vertical blue line in each sub graph) for periodic, chaotic and healthy RR interval time series respectively. These values increased by 0.0804, 0.0665 and 0.0266 respectively using DistEn measure, i.e., considering lags from 1:98. The increase in entropy values due to the addition of elements corresponding to lags over 10 was negligible compared to the already attained values from the first 10 lags.

This supports our hypothesis that the entropy of underlying physiological mechanism can be captured from a change of the signal of up to 10 lags rather than using all lags based on data length. Another benefit of using maximum lag as 10 is it reduces computational cost from $O(N^{2})$ to O(N). From Equation (3) it is obvious that for any data length *N* the number of elements to be calculated is $\left(N-m\right)\left(N-m-1\right)\approx O\left(N^{2}\right)$. On the other hand, for mDistEn the number of elements in $D^{{}^{\prime \prime }}$ is 10(N−m)≈O(N). Therefore, mDistEn reduces the computational burden and is suitable for energy constrained devices such as mobile or sensor devices.

### 3.2. mDistEn as a Classification Feature: Comparison with DistEn

**Figure 5:**
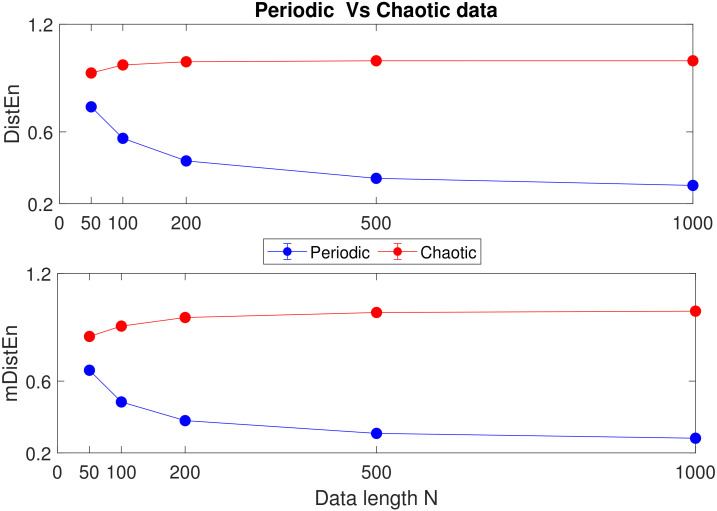
Periodic vs. chaotic data: mean±SD values of DistEn and mDistEn.

**Figure 6:**
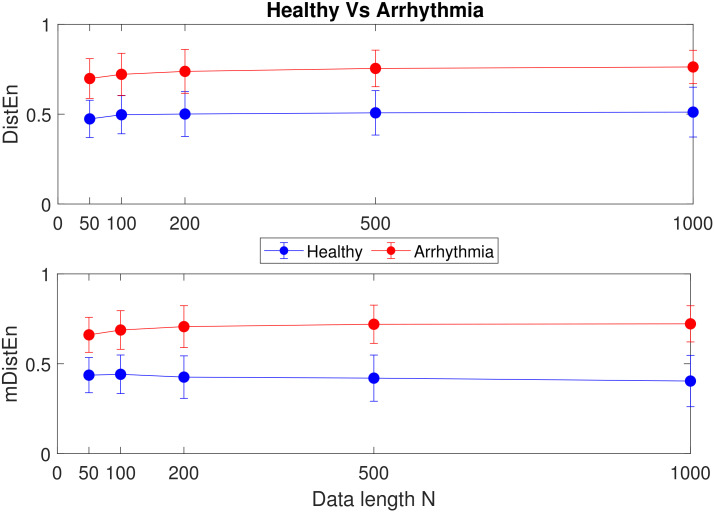
Healthy vs. arrhythmic HRV data: mean±SD values of DistEn and mDistEn.

**Figure 7:**
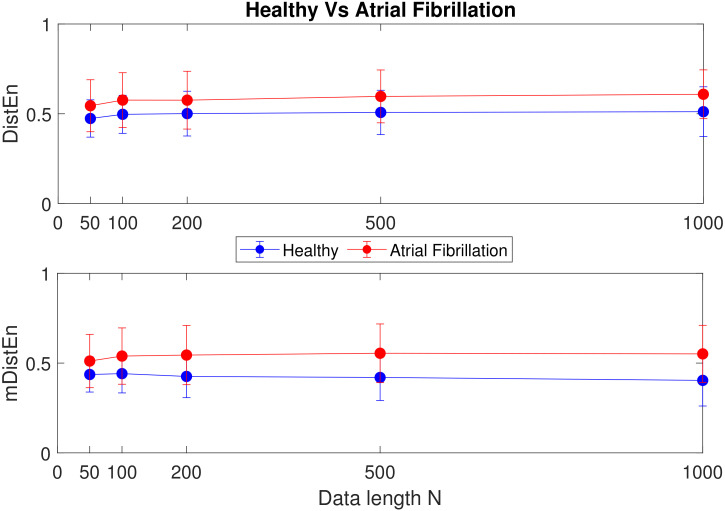
Healthy vs. atrial fibrillation HRV data: mean±SD values of DistEn and mDistEn.

The mean±SD values of DistEn and mDistEn corresponding to synthetic and physiological data are shown in Figure 5, Figure 6 and Figure 7. It can be seen that both the measures classify synthetic data very significantly and consistently across data length *N*, while for the physiological data, the significance of classification varies with data length *N*. A better sense of the classification can be gotten by calculating the corresponding *p*-values of significance (listed in Table 1). As can be seen from the table, for (a) the healthy vs. arrhythmic case, both DistEn and mDistEn classify the data set significantly at all data lengths. The significance is slightly more (smaller *p*-values) in the case of mDistEn. On the other hand, for (b) the healthy vs. atrial fibrillation case, DistEn shows significant classification only at the higher data lengths (N≥500). However, mDistEn shows significant classification from *N* as low as 100. Thus, mDistEn is surely better than DistEn at handling shorter lengths of data.

**Figure 8:**
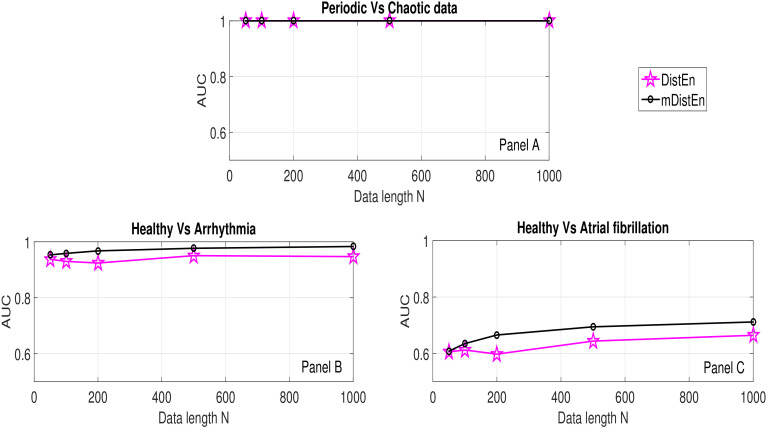
AUC values of DistEn and mDistEn in classification of data at various data lengths.

For further clarity here, the AUC values of DistEn and mDistEn corresponding to synthetic and physiological data are shown in Figure 8 and tabulated in Table 2. For synthetic signals, the AUC values of both mDistEn and DistEn are the same and consistent with respect to data length *N*. This shows that mDistEn performs equally to DistEn and supports the previous finding that DistEn is less affected by data length [20].

Looking at healthy vs. arrhythmia data, the AUC values of mDistEn are higher than those of DistEn and consistent with data length *N*. Therefore, mDistEn performs better than DistEn for all *N* and this improvement can be attributed to physiologically motivated selection of lags for evaluation of change in mDistEn measurement. Similarly, for healthy vs. atrial fibrillation data the AUC values show that mDistEn performs better than DistEn for all N≥100. At the lowest used data length of 50, the performances of the two methods are equal and not significant (NS). Overall, the results indicate that increasing lags in DistEn (with increasing data length) negatively affects the classification performance, which is avoided in mDistEn by choosing physiologically relevant number of lags.

## 4. Discussion

Complexity analysis of HRV signals has significant prognostic value. It could be used as an important non-invasive predictor of adverse cardiovascular events, such as arrhythmia and atrial fibrillation [28,29,30]. Many non-linear algorithms have been used to assess HRV complexity, especially the entropy methods [31]. Among these, DistEn is a recently introduced measure that is less parametric compared to traditional entropy formulations such as ApEn and SampEn [16].

Different methods capture one or several different aspects of signal complexity, including irregularity and fractal dynamics. DistEn captures irregularity of spatial structures (of a given time-series) in the state space that is unique for different dynamics [16].This represents one aspect of signal complexity. If, on the other hand we are interested in a measure of randomness, DistEn may not show the differentiation of a signal from its surrogate. However, this is true only when the surrogate data are generated by random shuffling of the original time series, not for surrogate data based on phase randomization. DistEn relies on the distribution of inter-vector distances that is retained theoretically after random shuffling but perturbed by other randomization processes. We may also interpret that DistEn appears sensitive to the irregularity of signal dynamics since it goes up as the number of random dynamics increases in the MIX process. This concept is in keeping with the two well-studied entropy ancestors ApEn and SampEn [16]. Thus, DistEn is not a complete measure of signal complexity and captures just a few aspects of it, each interpreted independently. In this study, we interpret complexity as the irregularity of spatial structures in the state space.

DistEn is an algorithm that focuses particularly on short-term data [16,20]. The idea behind DistEn is to map length-N RR intervals to an inter-vector distance matrix of dimension (N−m+1)×(N−m+1) in the state space. This logarithmically expands the limited information contained in the original RR interval time-series [16]. Examinations on both bench mark synthetic and real clinical data have indicated significantly improved stability and reliability of DistEn [16,20] over traditional methods. This is because DistEn uses the probability distribution of the entire inter-vector distance matrix; a global quantification as compared to the partial quantification seen in ApEn or SampEn [16].

In the present study, we have mapped inter-vector distances to the given RR intervals, using a limited time lag. In other words, we have reformed the estimation procedure of inter-vector distances in the original DistEn algorithm. The reformation was reminiscent of the possibility of not all elements in the distance matrix being physiologically significant. This is because the influence of a heartbeat may last until only 6–10 beats following it [26,27]. A modified DistEn (mDistEn) algorithm has been developed accordingly to restrict the time lag to a fixed value, thereby counting only those that are physiologically relevant to the template vector.

Our simulation tests on logistic and RR interval time series suggest that the proposed mDistEn (using only lags up to 10) accounts for ~90% (the ratios of mDtistEn/DistEn in Figure 2, Figure 3 and Figure 4 are close to 0.9) of what DistEn (using all possible lags) measures. This only indicates that the vectors corresponding to time lags > 10 contribute to a very small portion (less than 10%) of DistEn quantified information. Our tests also prove that the information captured by mDistEn (∼90%DistEn) has sufficient prognostic value to classify distinct data sets—in fact, more than that of DistEn. We have shown that mDistEn is a better classification feature than DistEn in differentiating arrhythmic or atrial fibrillation patients from healthy controls. Using physiologically insignificant lags (as DistEn does) only increases computational expense, adding absolutely no informative value. Consequently, a big advantage of our limited-lag algorithm is the reduction of computational complexity, giving it the potential to be embedded in modern, battery-driven wearable devices that are becoming increasingly popular these days.

An interesting question here would be about the role of the inter-vector distances corresponding to the larger lags (lags > 10). These appear to be largely negligible when comparing the absolute difference between DistEn and mDistEn. Looking from a physiological perspective, we understand that vagal and sympathetic mediation on RR intervals happen through the synaptic release of acetylcholine and noradrenaline, respectively. The vagal effects are almost immediate on a beat-by-beat basis as the turnover rate of acetylcholine is high. On the contrary, the noradrenaline is reabsorbed and metabolized relatively slowly, which results in a long effect latency of sympathetic mediation [32]. Therefore, it may seem necessary to use larger lags in entropy measurement (DistEn). However, the negligible difference between mDistEn and DistEn in presented scenarios clearly showed that most of the information can be captured with lag=(1…10). In this study, we have not used RR time series of very long durations such as ≥24 h, and therefore, the impact of very long duration HRV time series on the proposed mDistEn is currently unknown. This is a limitation of the current study and future exploration on continuous data from ambulatory monitoring could bring more light to the use of mDistEn for analyzing long-term HRV time series. For physiological signal other than HRV, a respective physiological mechanism should be considered to find the memory effect for determining range of lag. Therefore, we propose this modification to DistEn only for HRV analysis.

A second limitation of our study is that mDistEn was proposed in the context of HRV complexity analysis, after we had prior knowledge of the possible effect time (6–10 subsequent beats). Given a completely different data set to study (e.g., EEG data), mDistEn cannot be used unless there are clear implications on the restriction of effect time pertaining to the data. On the other hand, the original DistEn algorithm can still be used, irrespective of the data that are picked.

In conclusion, the better performance indicated by mDistEn in the current study does imply that in future, the design of algorithms could take "physiological context" into consideration too, in order for better accuracy and reduced computation, thereby maximizing the benefits of such algorithms.

## 5. Conclusions

This study examined distribution entropy (DistEn) measurement on HRV signal and modified the method to better reflect the complexity of underlying physiological mechanisms. We explained what the inter-vector distances in DistEn represent, when mapped to the given RR interval time series. DistEn uses multiple time lags to measure the Shannon entropy of changes in HRV signal. In this paper, we propose modified distribution entropy (mDistEn), a physiologically significant alternative to DistEn for HRV complexity analysis. Our experiments and analyses indicate that in comparison to DistEn, mDistEn could reduce computational costs and perform better in classifying both synthetic and physiological signals. Thus, mDistEn is a more pragmatic option over DistEn since it is (i) physiologically more relevant, (ii) computationally less expensive and (iii) a better classification feature, for HRV complexity analysis. 

## Figures and Tables

**Table 1:** *p* values of DistEn and mDistEn in classification of data at various data lengths.

*p*-Value										
	DistEn					mDistEn				
*N*	50	100	200	500	1000	50	100	200	500	1000
Periodic vs. Chaotic	1.59 × 10^−5^	1.59 × 10^−5^	1.59 × 10^−5^	1.59 × 10^−5^	1.59 × 10^−5^	1.59 × 10^−5^	1.59 × 10^−5^	1.59 × 10^−5^	1.59 × 10^−5^	1.59 × 10^−5^
Healthy vs. Arrhythmic	5.64 × 10^−16^	1.75 × 10^−15^	4.14 × 10^−15^	7.56 × 10^−17^	1.30 × 10^−16^	5.02 × 10^−17^	2.10 × 10^−17^	4.97 × 10^−18^	1.09 × 10^−18^	3.78 × 10^−19^
Healthy vs. Atrial Fibrillated	NS	NS	NS	0.03	0.01	NS	0.05	0.01	0.004	0.002

**Table 2:** AUC values of DistEn and mDistEn in classification of data at various data lengths.

	AUC									
	DistEn					mDistEn				
*N*	50	100	200	500	1000	50	100	200	500	1000
Periodic vs. Chaotic	1	1	1	1	1	1	1	1	1	1
Healthy vs. Arrhythmic	0.94	0.93	0.92	0.95	0.95	0.95	0.96	0.97	0.98	0.98
Healthy vs. Atrial Fibrillated	0.61	0.61	0.60	0.64	0.66	0.61	0.64	0.67	0.69	0.71
